# Monocyte chemotactic protein-1 deficiency attenuates and high-fat diet exacerbates bone loss in mice with Lewis lung carcinoma

**DOI:** 10.18632/oncotarget.15055

**Published:** 2017-02-03

**Authors:** Lin Yan, Forrest H. Nielsen, Sneha Sundaram, Jay Cao

**Affiliations:** ^1^ U.S. Department of Agriculture, Agricultural Research Service, Grand Forks Human Nutrition Research Center, Grand Forks, ND 58202, USA

**Keywords:** MCP-1, high-fat diet, bone, metastasis, mice

## Abstract

Bone loss occurs in obesity and cancer-associated complications including wasting. This study determined whether a high-fat diet and a deficiency in monocyte chemotactic protein-1 (MCP-1) altered bone structural defects in male C57BL/6 mice with Lewis lung carcinoma (LLC) metastases in lungs. Compared to non-tumor-bearing mice, LLC reduced bone volume fraction, connectivity density, trabecular number, trabecular thickness and bone mineral density and increased trabecular separation in femurs. Similar changes occurred in vertebrae. The high-fat diet compared to the AIN93G diet exacerbated LLC-induced detrimental structural changes; the exacerbation was greater in femurs than in vertebrae. Mice deficient in MCP-1 compared to wild-type mice exhibited increases in bone volume fraction, connectivity density, trabecular number and decreases in trabecular separation in both femurs and vertebrae, and increases in trabecular thickness and bone mineral density and a decrease in structure model index in vertebrae. Lewis lung carcinoma significantly decreased osteocalcin but increased tartrate-resistant acid phosphatase 5b (TRAP 5b) in plasma. In LLC-bearing mice, the high-fat diet increased and MCP-1 deficiency decreased plasma TRAP 5b; neither the high-fat diet nor MCP-1 deficiency resulted in significant changes in plasma concentration of osteocalcin. In conclusion, pulmonary metastasis of LLC is accompanied by detrimental bone structural changes; MCP-1 deficiency attenuates and high-fat diet exacerbates the metastasis-associated bone wasting.

## INTRODUCTION

Metastasis is the most devastating aspect of cancer, which is accompanied by wasting that eventually results in cachexia characterized by a significant skeletal muscle loss and multi-organ functional failures. Limited studies indicate that bone loss can occur during cancer-associated wasting. For example, lung cancer patients with a 30% loss of body mass exhibit significantly lower total body mineral content than healthy-matched controls [1]. Concurrent muscular and bone deterioration is found in a murine model of cancer cachexia [2]. Furthermore, pathways that induce muscular wasting may promote bone loss during cachexia [3].

Monocyte chemotactic protein-1 (MCP-1) is primarily considered a potent chemotactic factor that attracts monocytes and other inflammatory cells to the site of inflammation during tissue injury and infection [4]. However, MCP-1 has functions beyond tissue repair; it participates in the development and progression of many pathophysiological conditions, including cancer [5–7]. Elevated expression of MCP-1 is associated with poor outcomes and short disease-free intervals [5–7], and thus it has prognostic value for cancer patients. In support of the clinical observations, animal studies show that MCP-1 participates in primary tumor growth and metastasis [8–10]. In addition, MCP-1 is pro-osteoclastogenic. High expression of MCP-1 is found in osteoporotic bone of humans [11]*. In vitro* studies show that MCP-1 stimulates the formation of osteoclasts [12].

Adipose tissue produces proinflammatory cytokines including MCP-1. Adipose tissue expression of MCP-1 mRNA correlates positively with adiposity and body mass index [13]. Accumulation of visceral fat mass is an indicator of detrimental health outcomes in obesity [14, 15]. Being obese at the time of primary cancer diagnosis is predictive of poor prognosis [16–19]. Increased body weight caused by obesity was considered a benefit to bone health because of reported positive correlations between body weight and bone mineral density [20]. However, studies also show an inverse relationship between body fat mass and bone mass in humans [21]. Laboratory studies show that consumption of a high-fat diet results in bone loss in rodent models [22–24].

In our study of the relationship between obesity and metastasis, we found that consumption of a high-fat diet enhances and MCP-1 deficiency reduces spontaneous pulmonary metastasis of Lewis lung carcinoma (LLC) in mice [25]. Furthermore, we found bone loss in mice with lung metastases of LLC [24]. Thus, we hypothesized that a high-fat diet exacerbates and MCP-1 deficiency alleviates metastasis-associated bone wasting. To test this hypothesis, we performed micro-computed tomographic analysis of femurs and vertebrae collected from LLC-bearing wild-type and MCP-1^−/−^ mice in a previous study [25] showing that the high-fat diet enhances and MCP-1 deficiency attenuates metastasis of LLC.

## RESULTS

### Physical measurement of bone

There were no differences in femur length, medial-lateral axis width and anterior-posterior axis width between non-tumor-bearing and LLC-bearing wild-type mice fed the AIN93G diet (data not shown). In LLC-bearing mice, the femur length of MCP-1^−/−^ mice was slightly but significantly shorter than that of wild-type mice (14.53 ± 0.08 vs. 14.82 ± 0.08 mm, *p*<0.05); there were no differences in medial-lateral axis width and anterior-posterior axis width between MCP-1^−/−^ and wild-type mice (data not shown). The high-fat diet did not change these variables compared to the AIN93G diet (data not shown).

### Micro-computed tomographic measurement of trabecular bone

**Figure 1:**
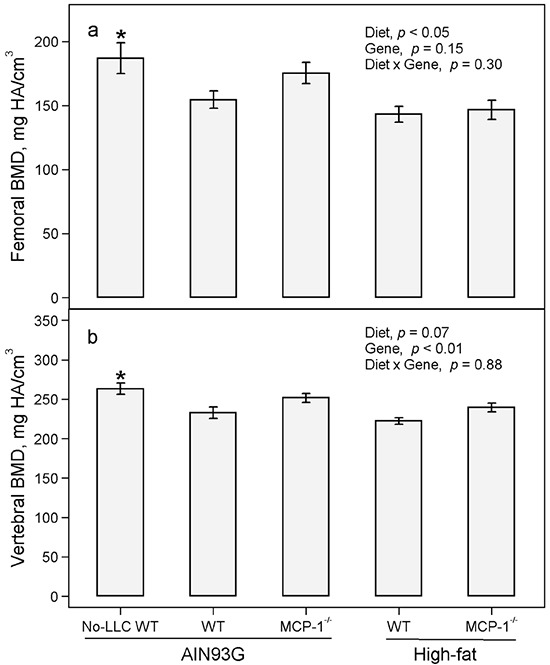
Bone mineral density of femurs and vertebrae of MCP-1^−/−^ and wild-type mice fed the AIN93G or the high-fat diet Two-way ANOVA were performed to compare differences among the groups of LLC-bearing mice. *A priori* contrasts were performed to compare differences between AIN93G-fed wild-type (WT) mice with or without Lewis lung carcinoma (No-LLC); *p<0.05 compared to AIN93G WT. Values are means ± SEM (n = 14-15 per group). BMD: bone mineral density.

The presence of LLC was detrimental to the three-dimensional microstructure of trabecular bone. Compared to non-tumor-bearing mice, LLC reduced bone volume fraction (BV/TV) by 29%, connectivity density (Conn.D) by 31%, trabecular number (Tb.N) by 11%, and trabecular thickness (Tb.Th) by 10% and increased trabecular separation (Tb.Sp) by 14% in femurs (Table 1). In lumbar vertebra 4, LLC reduced BV/TV by 19% and Tb.Th by 10% and increased structure model index (SMI) by 30% and Tb.Sp by 6% (Table 2). Furthermore, LLC reduced bone mineral density (BMD) by 17% in femurs (Figure 1a) and 12% in vertebrae (Figure 1b). Pearson correlation analysis showed that the volume of lung metastases correlated inversely with BV/TV, Conn.D, Tb.N and BMD and positively with SMI and Tb.Sp in both femurs and vertebrae (Table 3).

**Table 1:** Trabecular structural changes in femurs of MCP-1^−/−^ and wild-type mice fed the AIN93G or the high-fat diet

	AIN93GWild-type No LLC	AIN93GWild-type	AIN93GMCP-1^−/−^	High-fatWild-type	High-fatMCP-1^−/−^	Diet	*p* valuesGene	D × G
BV/TV, %	14.8 ± 1.0 *	10.5 ± 0.8	12.7 ± 0.9	9.8 ± 0.6	10.8 ± 0.5	0.10	< 0.05	0.41
Conn.D, 1/mm^3^	156.4 ± 12.9 *	108.3 ± 12.9	144.2 ± 12.0	90.3 ± 7.5	109.4 ± 8.4	< 0.05	< 0.05	0.44
SMI	2.2 ± 0.1	2.6 ± 0.1	2.4 ± 0.1	2.6 ± 0.1	2.5 ± 0.1	0.22	0.12	0.37
Tb.N, 1/mm	5.4 ± 0.1 *	4.8 ± 0.1	5.3 ± 0.1	4.5 ± 0.1	4.8 ± 0.1	< 0.01	< 0.01	0.30
Tb.Th, μm	46.5 ± 1.0 *	41.9 ± 0.6	42.6 ± 1.1	43.5 ± 0.8	43.3 ± 0.7	0.19	0.76	0.63
Tb.Sp, μm	181.5 ± 4.1 *	207.0 ± 6.3	186.0 ± 4.0	219.4 ± 5.3	206.9 ± 3.6	< 0.01	< 0.01	0.38

Two-way ANOVA was performed to compare differences among the groups of LLC-bearing mice. *A priori* contrasts were performed to compare the difference between AIN93G-fed wild-type mice with or without Lewis lung carcinoma (LLC);

*p<0.01 compared to AIN93G wild-type. Values are means ± SEM (n = 14-15 per group). BV/TV: bone volume fraction; Conn.D: connectivity density; SMI: structure model index; Tb.N: trabecular number; Tb.Th: trabecular thickness; Tb.Sp: trabecular separation.

**Table 2:** Trabecular structural changes in vertebrae of MCP-1^−/−^ and wild-type mice fed the AIN93G or the high-fat diet

	AIN93GWild-type No LLC	AIN93GWild-type	AIN93GMCP-1^−/−^	High-fatWild-type	High-fatMCP-1^−/−^	Diet	*p* valuesGene	D × G
BV/TV, %	23.7 ± 0.9 *	19.3 ± 1.3	22.1 ± 0.8	18.4 ± 0.5	20.8 ± 0.7	0.21	< 0.01	0.79
Conn.D, 1/mm^3^	267.2 ± 5.8	271.0 ± 8.1	297.1 ± 7.8	260.9 ± 7.0	283.1 ± 9.0	0.12	< 0.01	0.80
SMI	1.0 ± 0.1 *	1.3 ± 0.1	1.2 ± 0.10	1.5 ± 0.1	1.3 ± 0.1	0.05	< 0.05	0.54
Tb.N, 1/mm	5.7 ± 0.1	5.6 ± 0.1	5.7 ± 0.1	5.5 ± 0.1	5.6 ± 0.1	0.19	< 0.05	0.68
Tb.Th, μm	47.5 ± 1.0 *	42.8 ± 0.9	44.8 ± 0.7	42.7 ± 0.6	44.1 ± 0.9	0.65	< 0.05	0.69
Tb.Sp, μm	166.6 ± 1.7 *	175.7 ± 2.5	166.0 ± 2.1	177.2 ± 2.1	172.7 ± 2.6	0.07	< 0.01	0.25

Two-way ANOVA was performed to compare differences among the groups of LLC-bearing mice. *A priori* contrasts were performed to compare the difference between AIN93G-fed wild-type mice with or without Lewis lung carcinoma (LLC);

*p<0.01 compared to AIN93G wild-type. Values are means ± SEM (n = 14-15 per group). BV/TV: bone volume fraction; Conn.D: connectivity density; SMI: structure model index; Tb.N: trabecular number; Tb.Th: trabecular thickness; Tb.Sp: trabecular separation.

**Table 3:** Pearson correlations of the extent of lung metastasis with trabecular structural changes in femurs and vertebrae of MCP-1^−/−^ and wild-type mice fed the AIN93G or the high-fat diet

	Femurs				Vertebrae			
	Number of metastasis		Volume of metastasis		Number of metastasis		Volume of metastasis	
	*r*	*p*	*r*	*p*	*r*	*p*	*r*	*p*
BV/TV, %	−0.18	0.20	−0.48	< 0.01	−0.14	0.32	−0.40	< 0.01
Conn.D, 1/mm^3^	−0.21	0.13	−0.54	< 0.01	−0.20	0.17	−0.32	< 0.05
SMI	0.09	0.52	0.43	< 0.01	0.25	0.07	0.41	< 0.01
Tb.N, 1/mm	−0.30	< 0.05	−0.60	< 0.01	−0.14	0.34	−0.33	< 0.05
Tb.Th, μm	0.02	0.92	−0.07	0.60	−0.13	0.38	−0.34	< 0.05
Tb.Sp, μm	0.28	< 0.05	0.60	< 0.01	0.13	0.38	0.33	< 0.05
BMD, mg HA/cm^3^	−0.23	0.11	−0.51	< 0.01	−0.23	0.11	−0.46	< 0.01

Abbreviations: BV/TV: bone volume fraction; Conn.D: connectivity density; SMI: structure model index; Tb.N: trabecular number; Tb.Th: trabecular thickness; Tb.Sp: trabecular separation; BMD: bone mineral density.

The high-fat diet exacerbated the detrimental structural changes in trabecular bone mediated by LLC. Consuming the high-fat diet instead of the AIN93G diet decreased Conn.D by 21%, Tb.N by 8% and increased Tb.Sp by 9% in femurs (Table 1). The high-fat diet instead of the AIN93G diet increased SMI by 12% in vertebrae (Table 2). The high-fat diet decreased BMD by 12% in femurs (Figure 1a) but only marginally reduced BMD in vertebrae (Figure 1b).

Deficiency in MCP-1 attenuated detrimental structural changes in trabecular bone induced by LLC. The MCP-1 deficiency increased BV/TV by 16%, Conn.D by 28%, Tb.N by 9% and decreased Tb.Sp by 8% in femurs (Table 1). In vertebrae, MCP-1 deficiency increased BV/TV by 14%, Conn.D by 9%, Tb.N by 2% and Tb.Th by 4% and decreased SMI by 11% and Tb.Sp by 4% (Table 2). Deficiency in MCP-1 increased BMD in vertebrae by 8% (Figure 1b) but not in femurs (Figure 1a).

### Micro-computed tomographic measurement of cortical bone

Compared to non-tumor-bearing mice, LLC-bearing mice exhibited decreases of 4% and 6% in cortical area fraction (Ct.Ar/Tt.Ar) and 7% and 9% in cortical thickness (Ct.Th) in femur mid-shaft and vertebrae, respectively (Table 4). The high-fat diet did not change LLC-induced decreases in Ct.Ar/Tt.Ar and Ct.Th in either femurs or vertebrae (Table 4). Deficiency in MCP-1 resulted in a slight but significant increase in Ct.Ar/Tt.Ar in femurs but not in vertebrae, nor did it increase Ct.Th in either femurs or vertebrae (Table 4).

**Table 4:** Cortical structural changes in femurs and vertebrae of MCP-1^−/−^ and wild-type mice fed the AIN93G or the high-fat diet

	AIN93GWild-type No LLC	AIN93GWild-type	AIN93GMCP-1^−/−^	High-fatWild-type	High-fatMCP-1^−/−^	Diet	*p* valuesGene	D × G
Femurs								
Ct.Ar/Tt.Ar, %	47.1 ± 0.6 *	45.4 ± 0.6	45.7 ± 0.4	44.6 ± 0.5	46.5 ± 0.4	0.99	< 0.05	0.10
Ct. Th, μm	165.4 ± 2.2 *	154.6 2.7	153.4 ± 1.9	154.7 ± 1.5	156.1 ± 3.0	0.54	0.98	0.58
Vertebrae								
Ct.Ar/T.Ar, %	76.2 ± 0.7 *	71.8 ± 1.4	73.2 ± 0.8	72.3 ± 0.9	71.5 ± 1.0	0.55	0.76	0.27
Ct.Th, μm	47.1 ± 0.9	42.7 ± 0.9	43.6 ± 0.9	42.6 ± 0.6	43.4 ± 0.6	0.86	0.29	0.93

Two-way ANOVA was performed to compare differences among the groups of LLC-bearing mice. *A priori* contrasts were performed to compare differences between AIN93G-fed wild-type mice with or without Lewis lung carcinoma (LLC);

*p<0.01 compared to AIN93G wild-type. Values are means ± SEM (n = 14-15 per group). Ct.Ar/Tt.Ar: cortical area fraction; Ct.Th: cortical thickness.

### Concentrations of osteocalcin and tartrate-resistant acid phosphatase 5b (TRAP 5b) in plasma

**Figure 2:**
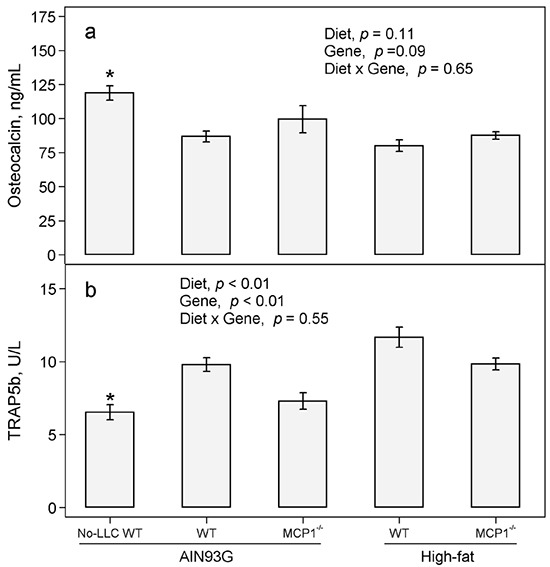
Plasma concentrations of osteocalcin and TRAP 5b in MCP-1^−/−^ and wild-type mice fed the AIN93G or the high-fat diet Two-way ANOVA were performed to compare differences among the groups of LLC-bearing mice. *A priori* contrasts were performed to compare differences between AIN93G-fed wild-type (WT) mice with or without Lewis lung carcinoma (No-LLC); *p<0.05 compared to AIN93G WT. Values are means ± SEM (n = 12 per group).

Compared to non-tumor bearing mice, LLC resulted in a 27% decrease in osteocalcin (Figure 2a) and a 50% increase in TRAP 5b in plasma (Figure 2b). There was no difference in osteocalcin between the high-fat and AIN93G diets and between MCP-1^−/−^ and wild-type mice (Figure 2a). The high-fat diet compared to the AIN93G diet increased TRAP 5b by 26% (Figure 2b); MCP-1 deficiency decreased TRAP 5b by 20% compared to wild-type mice (Figure 2b).

## DISCUSSION

The present study showed that the presence of LLC and its pulmonary metastases deteriorated trabecular and cortical structure and reduced bone mineral density in mice, indicating the significance of malignancy in bone wasting. The high-fat diet, which leads to increases in adipose MCP-1 [25], exacerbated and deficiency in MCP-1 attenuated LLC-mediated bone deterioration. These findings indicate that MCP-1 contributes to cancer-associated bone loss.

Lewis lung carcinoma metastasizes to lungs from a subcutaneous primary tumor [25, 26]. The present study showed that the extent of LLC metastasis correlated positively with the severity of bone loss in mice. The metastasis of LLC is accompanied by elevations in plasma concentrations of proinflammatory cytokines, e.g. MCP-1, plasminogen activator inhibitor-1 (PAI-1), tumor necrosis factor-α (TNF-α) [25, 27]. Available studies show that proinflammatory cytokines participate in bone homeostasis. For example, PAI-1 deficiency attenuates bone loss in estrogen-deficient [28] and diabetic mice [29] and in LLC-bearing mice [24]; TNF-α promotes osteoclastogenesis and osteoclast activation [30, 31]. While exact mechanisms through which LLC reduces bone mass remain to be elucidated, our results indicate that increases in production of proinflammatory cytokines may contribute to bone loss.

This study provides evidence that MCP-1 contributes positively to the bone loss associated with cancer metastasis. This finding is supported by reports that MCP-1 is highly expressed at sites of osteoporotic bone [11], prostate cancer-induced bone resorption [32] and bacteria-induced bone loss [33]. Furthermore, *in vitro* studies show that MCP-1 stimulates osteoclast formation [12, 34] and that lack of MCP-1 decreases the numbers and activity of osteoclasts *in vitro* and elevates bone mass in mice [35].

Adipose tissue, considered an endocrine organ, produces inflammatory cytokines. In the present study, the high-fat diet increased body adiposity and plasma concentrations of all proinflammatory cytokines quantified including MCP-1 (not in MCP-1^−/−^ mice), PAI-1, TNF-α and leptin [25]. As aforementioned, these cytokines participate in bone metabolism, and their elevations often result in bone loss [28–31, 35] and osteoclastogenesis [30, 31]. Thus, elevations of these cytokines likely contributed to the high-fat diet exacerbating bone loss in LLC-bearing mice.

Bone remodeling is a highly coordinated process of bone formation and bone resorption. Osteocalcin is considered an indicator of bone formation [36, 37]. Serum levels of osteocalcin are lower in lung cancer patients with bone metastasis compared to those with no or delayed metastasis [38, 39]. Mounting evidence show that TRAP 5b is a useful marker of bone resorption [40, 41]. Serum concentrations of TRAP 5b correlate positively with the extent of bone lesions in patients with multiple myeloma [42] and increase in breast cancer patients with skeletal metastasis [43]. In the present study, the decrease in plasma osteocalcin and the increase in TRAP 5b in LLC-bearing mice indicate that LLC metastasis may uncouple bone remodeling. This uncoupling may be responsible for the detrimental changes in bone structure and mineral density in those mice.

In LLC-bearing mice, neither the high-fat diet decreased nor MCP-1 deficiency increased the plasma concentrations of osteocalcin compared to their corresponding controls. Therefore, their observed effects on bone structure likely were not via bone formation. The finding that the high-fat diet increased plasma TRAP 5b suggests that the high-fat diet may uncouple bone remodeling by promoting bone resorption. This suggestion is supported by reports that elevated TRAP 5b in plasma [22, 44] is associated with obesity-induced bone loss. Our finding that MCP-1^−/−^ mice exhibited decreased plasma TRAP 5b suggests that MCP-1 deficiency may restore bone mass by reducing bone resorption. Furthermore, MCP-1 binds to the MCP-1 receptor expressed in osteoclasts [12] and deficiency in MCP-1 receptor results in higher bone mass in mice [45]. Thus, all these studies support our finding that MCP-1 may contribute to bone loss during LLC metastasis by promoting bone resorption but not by reducing bone formation.

In summary, the present study showed detrimental structural changes in trabecular and cortical bone and reduction in bone mineral density in mice with LLC and its pulmonary metastases. It indicates that LLC aggression leads to bone loss in this mouse model. Our findings support the clinical observations that cancer progression accompanies bone wasting [1] and disturbance of bone remodeling [38, 39, 42, 43]. Furthermore, it indicates the relevance and usefulness of this spontaneous metastasis model to study cancer-associated bone wasting. That feeding mice the high-fat diet exacerbated and MCP-1 deficiency attenuated LLC-mediated bone loss indicate that both high-fat diet and MCP-1 contribute positively to bone loss in metastasis-associated wasting. It suggests that approaches that lead to a reduction in cancer-promoting proinflammatory cytokine MCP-1, including that produced by the adipose tissue, may attenuate not only cancer progression but also its associated bone wasting.

## MATERIALS AND METHODS

### Animals and diets

Four to five-week old male MCP-1 deficient mice (MCP-1 ^−/−^, B6.129S4-*Ccl2^tm1Rol^*/J, C57BL/6J background) and C57BL/6J wild-type mice (The Jackson Laboratory, Bar Harbor, ME, USA) were housed in a pathogen-free room with a 12:12-hour light/dark cycle and maintained at 22 ± 1°C [25]. Mice were fed a modified AIN93G diet [46] providing 16% or 45% (high-fat diet) of energy from corn oil. Mice had free access to their diets and deionized water; they were weighed weekly. Both diets were powder diets; they were stored at -20°C until feeding.

### Lewis lung carcinoma cells

Lewis lung carcinoma cell line, a variant that metastasizes to lungs [26], was obtained from Dr. Pnina Brodt, McGill University, Montreal, Quebec, Canada. The cells were cultured with RPMI-1640 medium containing 10% heat-inactivated fetal bovine serum and maintained in a humidified atmosphere of 5% CO_2_ in air at 37°C. Cells used for metastasis studies were *in vivo*-selected once [47]. Cells were free of mycoplasma based on Hoechst DNA staining and direct culture tests performed by American Type Cell Collection (Manassas, VA, USA).

### Experimental design

The study was approved by the Institutional Animal Care and Use Committee of the U.S. Department of Agriculture, Agricultural Research Service, Grand Forks Human Nutrition Research Center. The procedures followed the guidelines of the National Institutes of Health for the care and use of laboratory animals [48]. The experimental design was reported previously [25]. Briefly, MCP-1^−/−^ (n = 21 per group) and wild-type mice (n = 28 per group) were fed their respective AIN93G or high-fat diet for 7 weeks before each mouse was subcutaneously injected with 2.5 × 10^5^ viable LLC cells in the lower dorsal region. In addition, a separate group of wild-type mice (n = 18) fed the AIN93G diet but not injected with LLC cells served as controls to compare changes in bone structure and related biomarkers to LLC-bearing wild-type mice fed the same AIN93G diet. The subcutaneous primary tumor was removed surgically 11 days later when it was approximately 1 cm in diameter. Following surgery, mice were maintained on their respective diets for an additional 10 days. At termination, mice were euthanized, and their lungs were removed for determination of the extent of metastasis [25]. The right femur and vertebral column (from 10^th^ or 11^th^ thoracic vertebra to sacrum) from each mouse was collected and stored in phosphate-buffered saline for micro-computed tomographic analysis of trabecular and cortical bone. Plasma was collected for quantifying osteocalcin and tartrate-resistant acid phosphatase 5b (TRAP 5b).

### Bone evaluation

Femurs were cleaned with cheese cloth for physical measurements before micro-computed tomographic analysis. Femoral length along the proximal distal direction and mid-shaft widths in both medial-lateral and anterior-posterior axes were measured by using a digital caliper (Fred V. Fowler Company, Newton, MA, USA). Femurs and lumbar vertebral bodies were evaluated for trabecular and cortical structural properties by using high-resolution (12-μm slice increment) micro-computed tomography (μCT-40; Scanco Medical, Basserdorf, Switzerland) with *x*-ray source power of 55 KeV and 145 μA and integration time of 300 ms. A fixed threshold of 275 was used to delineate mineralized bone from soft tissue and marrow phase. In distal femurs, trabecular bone was evaluated in 125 slices (1.5 mm) of the metaphysis proximal to the distal growth plate, and cortical bone was evaluated in 100 slices (1.2 mm) at mid-shaft of the femur. In vertebral bodies, trabecular and cortical bone were analyzed along the entire cranial-caudal axis of the 4^th^ lumbar vertebra. The evaluation followed guidelines for assessment of bone microstructure in rodents using micro-computed tomography [49].

For trabecular bone, total volume (TV, mm^3^), bone volume (BV, mm^3^), bone volume fraction (ratio of the segmented bone volume to the total volume of the region evaluated, BV/TV, %), connectivity density (a degree of connectivity of trabeculae normalized by TV, Conn.D, 1/mm^3^), structure model index (an indicator of the plate- and rod-like geometry of trabecular structure, SMI), trabecular number (the average number of trabeculae per unit length, Tb.N, 1/mm), trabecular thickness (mean thickness of trabeculae, Tb.Th, mm), trabecular separation (mean distance between trabeculae, Tb.Sp, mm) and bone mineral density (BMD, mg hydroyxapatite/cm^3^) were measured in distal femurs and vertebrae. For cortical bone, total cross-sectional area inside the periosteal envelope (Tt.Ar, mm^2^), cortical bone area (Ct.Ar, mm^2^), cortical area fraction (Ct.Ar/T.Ar, %) and average cortical thickness (Ct.Th, mm) were computed for the femoral mid-shaft and vertebrae.

### Quantification of osteocalcin and TRAP 5b in plasma

Concentrations of osteocalcin (Alpco Diagnostics, Salem, NH, USA) and TRAP 5b (Immunodiagnostic Systems, Scottsdale, AZ, USA) in plasma were quantified by using sandwich enzyme-linked immunosorbent assay kits following protocols provided by the manufacturers. Samples were read within the linear range of the assay; the accuracy of the analysis was confirmed by the controls provided in each assay kit.

### Statistical analyses

The effects of diet (AIN93G or high-fat), genotype (MCP-1^−/−^ or wild-type) and their interaction were tested by using two-way analysis of variance (ANOVA) and Tukey contrasts. To examine the effect of LLC on bone structural changes and plasma concentrations of osteocalcin and TRAP 5b, *a priori* contrasts were used to test for differences in AIN93G-fed wild-type mice with or without LLC. Pearson correlation analysis was performed to examine associations between the extent of lung metastasis and trabecular structural changes in femurs and vertebrae. All data are presented as means ± standard error of the mean (SEM). Differences with a *p*-value of 0.05 or less were considered significant. All analyses were performed by using SAS software (version 9.4, SAS Institute, Cary, NC, USA).

## References

[1] Preston T, Fearon KCH, Robertson I, East BW, Calman KC, Ellis KJ, Yasumura S, Morgan WD ((1987)). Tissue loss during severe wasting in lung cancer patients. In Vivo Body Composition Studies.

[2] Choi E, Carruthers K, Zhang L, Thomas N, Battaglino RA, Morse LR, Widrick JJ (2013). Concurrent muscle and bone deterioration in a murine model of cancer cachexia. Physiol Rep.

[3] Kandarian S (2008). The molecular basis of skeletal muscle atrophy - parallels with osteoporotic signaling. J Musculoskelet Neuronal Interact.

[4] Carr MW, Roth SJ, Luther E, Rose SS, Springer TA (1994). Monocyte chemoattractant protein 1 acts as a T-lymphocyte chemoattractant. Proc Natl Acad Sci U S A.

[5] Lu Y, Cai Z, Galson DL, Xiao G, Liu Y, George DE, Melhem MF, Yao Z, Zhang J (2006). Monocyte chemotactic protein-1 (MCP-1) acts as a paracrine and autocrine factor for prostate cancer growth and invasion. Prostate.

[6] Lebrecht A, Grimm C, Lantzsch T, Ludwig E, Hefler L, Ulbrich E, Koelbl H (2004). Monocyte chemoattractant protein-1 serum levels in patients with breast cancer. Tumour Biol.

[7] Yoshidome H, Kohno H, Shida T, Kimura F, Shimizu H, Ohtsuka M, Nakatani Y, Miyazaki M (2009). Significance of monocyte chemoattractant protein-1 in angiogenesis and survival in colorectal liver metastases. Int J Oncol.

[8] Fridlender ZG, Kapoor V, Buchlis G, Cheng G, Sun J, Wang LC, Singhal S, Snyder LA, Albelda SM (2011). Monocyte chemoattractant protein-1 blockade inhibits lung cancer tumor growth by altering macrophage phenotype and activating CD8+ cells. Am J Respir Cell Mol Biol.

[9] Nam JS, Kang MJ, Suchar AM, Shimamura T, Kohn EA, Michalowska AM, Jordan VC, Hirohashi S, Wakefield LM (2006). Chemokine (C-C motif) ligand 2 mediates the prometastatic effect of dysadherin in human breast cancer cells. Cancer Res.

[10] Bussard KM, Okita N, Sharkey N, Neuberger T, Webb A, Mastro AM (2010). Localization of osteoblast inflammatory cytokines MCP-1 and VEGF to the matrix of the trabecula of the femur, a target area for metastatic breast cancer cell colonization. Clin Exp Metastasis.

[11] Hopwood B, Tsykin A, Findlay DM, Fazzalari NL (2009). Gene expression profile of the bone microenvironment in human fragility fracture bone. Bone.

[12] Kim MS, Day CJ, Morrison NA (2005). MCP-1 is induced by receptor activator of nuclear factor-{kappa}B ligand, promotes human osteoclast fusion, and rescues granulocyte macrophage colony-stimulating factor suppression of osteoclast formation. J Biol Chem.

[13] Christiansen T, Richelsen B, Bruun JM (2005). Monocyte chemoattractant protein-1 is produced in isolated adipocytes, associated with adiposity and reduced after weight loss in morbid obese subjects. Int J Obes (Lond).

[14] Huber J, Kiefer FW, Zeyda M, Ludvik B, Silberhumer GR, Prager G, Zlabinger GJ, Stulnig TM (2008). CC chemokine and CC chemokine receptor profiles in visceral and subcutaneous adipose tissue are altered in human obesity. J Clin Endocrinol Metab.

[15] Bruun JM, Lihn AS, Pedersen SB, Richelsen B (2005). Monocyte chemoattractant protein-1 release is higher in visceral than subcutaneous human adipose tissue (AT): implication of macrophages resident in the AT. J Clin Endocrinol Metab.

[16] Daniell HW (1988). Increased lymph node metastases at mastectomy for breast cancer associated with host obesity, cigarette smoking, age, and large tumor size. Cancer.

[17] Loi S, Milne RL, Friedlander ML, McCredie MR, Giles GG, Hopper JL, Phillips KA (2005). Obesity and outcomes in premenopausal and postmenopausal breast cancer. Cancer Epidemiol Biomarkers Prev.

[18] Bassett WW, Cooperberg MR, Sadetsky N, Silva S, DuChane J, Pasta DJ, Chan JM, Anast JW, Carroll PR, Kane CJ (2005). Impact of obesity on prostate cancer recurrence after radical prostatectomy: data from CaPSURE. Urology.

[19] Amling CL, Riffenburgh RH, Sun L, Moul JW, Lance RS, Kusuda L, Sexton WJ, Soderdahl DW, Donahue TF, Foley JP, Chung AK, McLeod DG (2004). Pathologic variables and recurrence rates as related to obesity and race in men with prostate cancer undergoing radical prostatectomy. J Clin Oncol.

[20] Felson DT, Zhang Y, Hannan MT, Anderson JJ (1993). Effects of weight and body mass index on bone mineral density in men and women: the Framingham study. J Bone Miner Res.

[21] Zhao LJ, Liu YJ, Liu PY, Hamilton J, Recker RR, Deng HW (2007). Relationship of obesity with osteoporosis. J Clin Endocrinol Metab.

[22] Cao JJ, Gregoire BR, Gao H (2009). High-fat diet decreases cancellous bone mass but has no effect on cortical bone mass in the tibia in mice. Bone.

[23] Patsch JM, Kiefer FW, Varga P, Pail P, Rauner M, Stupphann D, Resch H, Moser D, Zysset PK, Stulnig TM, Pietschmann P (2011). Increased bone resorption and impaired bone microarchitecture in short-term and extended high-fat diet-induced obesity. Metabolism.

[24] Yan L, Nielsen FH, Sundaram S, Cao J (2015). High-fat diet enhances and plasminogen activator inhibitor-1 deficiency attenuates bone loss in mice with lewis lung carcinoma. Anticancer Res.

[25] Yan L, Sundaram S (2016). Monocyte chemotactic protein-1 deficiency reduces spontaneous metastasis of Lewis lung carcinoma in mice fed a high-fat diet. Oncotarget.

[26] Brodt P (1986). Characterization of two highly metastatic variants of Lewis lung carcinoma with different organ specificities. Cancer Res.

[27] Yan L, DeMars LC (2014). Effects of a high-fat diet on spontaneous metastasis of Lewis lung carcinoma in plasminogen activator inhibitor-1 deficient and wild-type mice. PLoS ONE.

[28] Daci E, Verstuyf A, Moermans K, Bouillon R, Carmeliet G (2000). Mice lacking the plasminogen activator inhibitor 1 are protected from trabecular bone loss induced by estrogen deficiency. J Bone Miner Res.

[29] Kawao N, Tamura Y, Okumoto K, Yano M, Okada K, Matsuo O, Kaji H (2013). Plasminogen plays a crucial role in bone repair. J Bone Miner Res.

[30] Fuller K, Murphy C, Kirstein B, Fox SW, Chambers TJ (2002). TNFalpha potently activates osteoclasts, through a direct action independent of and strongly synergistic with RANKL. Endocrinology.

[31] Lam J, Takeshita S, Barker JE, Kanagawa O, Ross FP, Teitelbaum SL (2000). TNF-alpha induces osteoclastogenesis by direct stimulation of macrophages exposed to permissive levels of RANK ligand. J Clin Invest.

[32] Lu Y, Cai Z, Xiao G, Keller ET, Mizokami A, Yao Z, Roodman GD, Zhang J (2007). Monocyte chemotactic protein-1 mediates prostate cancer-induced bone resorption. Cancer Res.

[33] Chae P, Im M, Gibson F, Jiang Y, Graves DT (2002). Mice lacking monocyte chemoattractant protein 1 have enhanced susceptibility to an interstitial polymicrobial infection due to impaired monocyte recruitment. Infect Immun.

[34] Miyamoto K, Ninomiya K, Sonoda KH, Miyauchi Y, Hoshi H, Iwasaki R, Miyamoto H, Yoshida S, Sato Y, Morioka H, Chiba K, Egashira K, Suda T, Toyama Y, Miyamoto T (2009). MCP-1 expressed by osteoclasts stimulates osteoclastogenesis in an autocrine/paracrine manner. Biochem Biophys Res Commun.

[35] Sul OJ, Ke K, Kim WK, Kim SH, Lee SC, Kim HJ, Kim SY, Suh JH, Choi HS (2012). Absence of MCP-1 leads to elevated bone mass via impaired actin ring formation. J Cell Physiol.

[36] Weinreb M, Shinar D, Rodan GA (1990). Different pattern of alkaline phosphatase, osteopontin, and osteocalcin expression in developing rat bone visualized by in situ hybridization. J Bone Miner Res.

[37] Boivin G, Morel G, Lian JB, Anthoine-Terrier C, Dubois PM, Meunier PJ (1990). Localization of endogenous osteocalcin in neonatal rat bone and its absence in articular cartilage: effect of warfarin treatment. Virchows Archiv.

[38] Terpos E, Kiagia M, Karapanagiotou EM, Charpidou A, Dilana KD, Nasothimiou E, Harrington KJ, Polyzos A, Syrigos KN (2009). The clinical significance of serum markers of bone turnover in NSCLC patients: surveillance, management and prognostic implications. Anticancer Res.

[39] Karapanagiotou EM, Terpos E, Dilana KD, Alamara C, Gkiozos I, Polyzos A, Syrigos KN (2010). Serum bone turnover markers may be involved in the metastatic potential of lung cancer patients. Med Oncol.

[40] Halleen JM, Alatalo SL, Suominen H, Cheng S, Janckila AJ, Vaananen HK (2000). Tartrate-resistant acid phosphatase 5b: a novel serum marker of bone resorption. J Bone Miner Res.

[41] Kirstein B, Chambers TJ, Fuller K (2006). Secretion of tartrate-resistant acid phosphatase by osteoclasts correlates with resorptive behavior. Cell Biochem.

[42] Terpos E, J de la Fuente, Szydlo R, Hatjiharissi E, Viniou N, Meletis J, Yataganas X, Goldman JM, Rahemtulla A (2003). Tartrate-resistant acid phosphatase isoform 5b: a novel serum marker for monitoring bone disease in multiple myeloma. Int J Cancer.

[43] Chao TY, Ho CL, Lee SH, Chen MM, Janckila A, Yam LT (2004). Tartrate-resistant acid phosphatase 5b as a serum marker of bone metastasis in breast cancer patients. J Biomed Sci.

[44] Yan L, Graef GL, Nielsen FH, Johnson LK, Cao J (2015). Soy protein is beneficial but high-fat diet and voluntary running are detrimental to bone structure in mice. Nutr Res.

[45] Binder NB, Niederreiter B, Hoffmann O, Stange R, Pap T, Stulnig TM, Mack M, Erben RG, Smolen JS, Redlich K (2009). Estrogen-dependent and C-C chemokine receptor-2-dependent pathways determine osteoclast behavior in osteoporosis. Nat Med.

[46] Reeves PG, Nielsen FH, Fahey GCJ (1993). AIN-93 purified diets for laboratory rodents: final report of the American Institute of Nutrition ad hoc writing committee on the reformulation of the AIN-76A rodent diet. J Nutr.

[47] Yan L, Demars LC (2010). Effects of dietary fat on spontaneous metastasis of Lewis lung carcinoma in mice. Clin Exp Metastasis.

[48] Institute for Laboratory Animal Research ((2011)). Guide for the Care and Use of Laboratory Animals.

[49] Bouxsein ML, Boyd SK, Christiansen BA, Guldberg RE, Jepsen KJ, Muller R (2010). Guidelines for assessment of bone microstructure in rodents using micro-computed tomography. J Bone Miner Res.

